# Large-bore aspiration of complex intra-abdominal abscesses using a mechanical aspiration device

**DOI:** 10.1007/s00261-025-05178-2

**Published:** 2025-08-23

**Authors:** Gavin Wu, Matthew Abad-Santos, David S. Shin, Eric J. Monroe, Jeffrey Forris Beecham Chick, Mina S. Makary

**Affiliations:** 1 Division of Vascular and Interventional Radiology, Department of Radiology, The Ohio State University Wexner Medical Center, Columbus, USA; 2 Section of Vascular and Interventional Radiology, Department of Radiology, University of Washington, Seattle, USA; 3 Division of Vascular and Interventional Radiology, Department of Radiology, University of Southern California, Los Angeles, USA; 4 Section of Vascular and Interventional Radiology, Department of Radiology, University of Wisconsin–Madison, Madison, USA

**Keywords:** Abdominal abscess, Intra-abdominal abscess, Percutaneous drainage, Mechanical aspiration, FlowTriever

## Abstract

**Purpose:**

To describe the use of a large-bore mechanical aspiration system (FlowTriever Aspiration Catheter) as an off-label intervention for the evacuation of complex intra-abdominal abscesses that were refractory to standard percutaneous drainage techniques.

**Methods:**

Two patients with large, complex intra-abdominal abscesses underwent aspiration using the FlowTriever Aspiration Catheter following failure of conventional catheter drainage. One patient had a walled-off necrotic collection from necrotizing pancreatitis; the other developed a postoperative abscess following appendectomy for perforated appendicitis. In each case, tract dilation was performed to accommodate the large-bore aspiration catheter, and follow-up CT imaging was used to assess treatment response.

**Results:**

In both cases, the FlowTriever Aspiration Catheter enabled high-flow aspiration of solid and purulent material, with successful placement of large-bore drainage catheters. Follow-up imaging demonstrated marked reduction or near-complete collapse of the abscess cavities. No procedure-related complications were observed.

**Conclusion:**

Large-bore mechanical aspiration with the FlowTriever Aspiration Catheter may offer an effective adjunct or alternative to catheter upsizing and fibrinolytic therapy in patients with complex intra-abdominal abscesses, particularly when standard drainage techniques are unsuccessful. This approach may reduce the need for repeat interventions or surgical debridement in appropriately selected patients.

**Editor**

Percutaneous catheter drainage remains the standard-of-care for managing intra-abdominal abscesses. However, large, complex collections with thick or necrotic debris often fail to respond adequately to this method, even after catheter upsizing or lavage. The FlowTriever Aspiration Catheter (Inari Medical Irvine, CA), originally developed for mechanical thrombectomy in deep vein thrombosis, has more recently been used for evacuating multiloculated hepatic abscesses unresponsive to conventional drainage [1]. The authors present two cases of complex intra-abdominal abscesses managed with the FlowTriever Aspiration Catheter, suggesting a potential role for this device in managing collections refractory to conventional percutaneous techniques.

These case reports were deemed exempt from Institutional review board (IRB) approval. A 73-year-old woman with necrotizing pancreatitis and septic shock presented with a 13.2 × 4.5 cm intra-abdominal collection on computed tomography (CT) consistent with walled-off necrosis and superinfection (Fig. 1a). Initial access was obtained under ultrasound guidance with an 18-gauge needle and 0.035-inch wire, and cone-beam CT was performed to confirm intraluminal catheter position within the complex walled-off collection. Aspiration was first attempted using a standard catheter; however, due to the viscosity of the contents and the complexity of the cavity, minimal output was obtained. A T24 FlowTriever Aspiration Catheter was then advanced into the collection (Fig. 1b). Four aspiration passes were performed, yielding solid debris and viscous purulent material. The aspiration catheter was subsequently exchanged for a 24-French drainage catheter (Fig. 1c). Aspirate sample analysis showed mixed bacterial, yeast, and food materials without evidence of malignancy. Follow-up CT performed four days later demonstrated substantial interval reduction in abscess size to 5.0 × 3.8 cm. No immediate procedure-related complications were observed. The patient declined any additional interventions and ultimately expired 15 days after drainage from multi-organ failure and cardiovascular collapse due to comorbidities unrelated to the procedure.

**Fig. 1:**
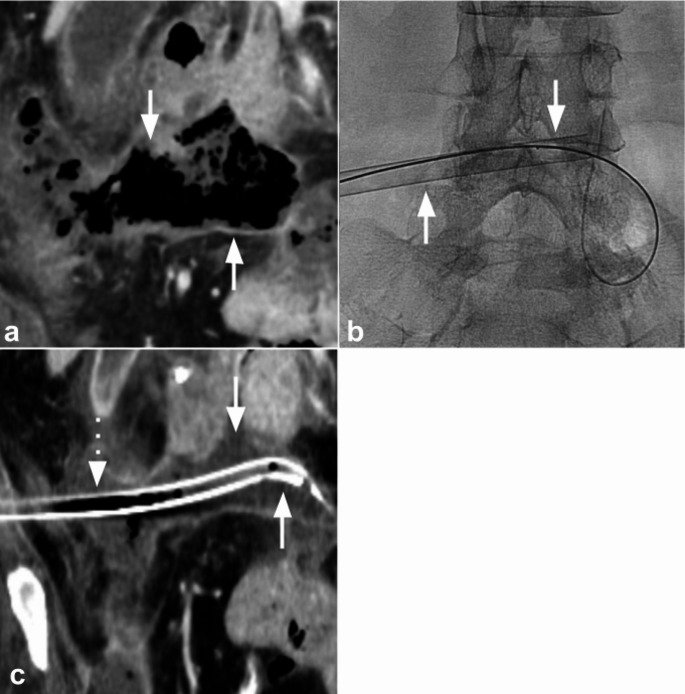
Large-bore aspiration of a walled-off necrotic collection using the FlowTriever Aspiration Catheter in a patient with necrotizing pancreatitis (a) Coronal contrast-enhanced computed tomography (CT) of the abdomen and pelvis shows a 13.2 × 4.5 cm collection consistent with walled-off necrosis (arrows) (b) Fluoroscopic image shows tract dilation and placement of the T24 FlowTriever Aspiration Catheter (arrows) (c) Follow-up contrast-enhanced CT of the abdomen and pelvis obtained four days later shows a reduction in abscess size to 5.0 × 3.8 cm (arrows) with a 24-French drainage catheter in place (dashed arrow)

A second case involved a 45-year-old man who presented with a postoperative intra-abdominal abscess following an appendectomy for perforated appendicitis. Imaging with contrast-enhanced CT revealed a 17.3 × 6.6 cm complex collection (Fig. 2a). Although he initially underwent placement of a 12-French drain upon admission, follow-up CT four days later showed minimal improvement, with a complex abscess measuring 12.9 × 5.4 cm. The drain was then upsized to 16-French, but follow-up CT demonstrated a persistent collection measuring 11.4 × 2.8 cm with limited drain output. Given the lack of adequate drainage and ongoing clinical infection, large-bore aspiration was performed, and the existing drain was removed over a wire and the tract dilated to advance a T20 Curve FlowTriever Aspiration Catheter into the abscess (Fig. 2b). Three large-bore aspirations were then performed and a 20-French drain was placed. The procedure was performed under general anesthesia at the patient’s request due to concern for potential discomfort. CT imaging the following day showed near-complete resolution of the abscess with cavity collapse around the drain (Fig. 2c). Follow-up imaging performed at routine post-procedure surveillance demonstrated complete resolution of the collection with no evidence of recurrence.

**Fig. 2:**
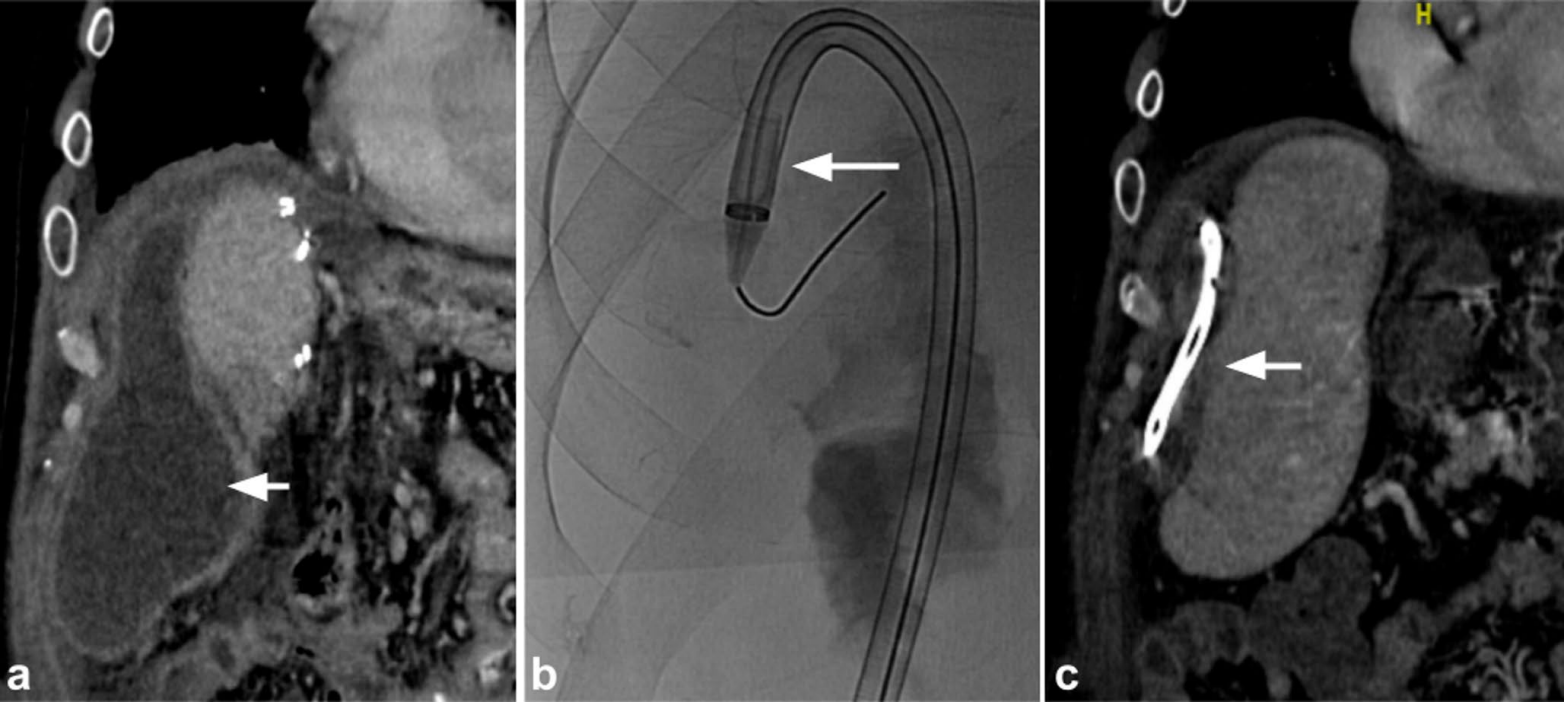
Large-bore aspiration of a postoperative intra-abdominal abscess using the FlowTriever Aspiration Catheter (a) Coronal contrast-enhanced computed tomography (CT) of the abdomen shows a 17.3 × 6.6 cm complex fluid collection following appendectomy for perforated appendicitis (arrow) (b) Fluoroscopic image shows the T20 FlowTriever Aspiration Catheter advanced into the collection (arrow) (c) Follow-up coronal contrast-enhanced CT of the abdomen and pelvis obtained one day later shows near-complete collapse of the cavity around the indwelling drain (arrow)

Complex intra-abdominal abscesses are associated with significant morbidity, including prolonged hospitalization, increased procedural burden, and higher healthcare costs [2]. While percutaneous catheter drainage remains the standard-of-care, collections containing necrotic debris or inspissated material may not respond adequately to conventional techniques such as catheter upsizing or fibrinolytic therapy [3]. The FlowTriever Aspiration Catheter’s steel-reinforced, large-bore aspiration catheters (16–24-French) enable high-flow aspiration without catheter collapse. While originally developed for thrombectomy, its mechanical capability makes it suitable for off-label use in managing infected or debris-laden collections. In our cases, its use enabled successful evacuation of complex abscesses that had failed standard catheter drainage. For instance, in our second case, the treated collection resolved completely with no recurrence on follow-up at routine surveillance. As such, this approach may reduce the need for repeated interventions or surgical debridement.

While these results are encouraging, long-term durability remains to be established. Future prospective, randomized trials are warranted. Considerations such as device cost and availability must also be weighed. While the FlowTriever Aspiration Catheter represents a substantially higher upfront expense compared with standard percutaneous drains or adjunctive fibrinolytic therapy, complex collections often require multiple follow-up procedures, prolonged drainage, additional imaging, and in some cases, surgical washout, all of which increase overall resource utilization. In our cases, rapid cavity collapse and clinical improvement potentially reduced the need for repeat interventions and prolonged hospital stays. Nonetheless, cost-effectiveness analyses are needed to weigh device costs against total care expenditures, particularly in resource-limited settings.

## Data Availability

No datasets were generated or analysed during the current study.
